# Stressor-Cortisol Concordance Among Individuals at Clinical High-Risk for Psychosis: Novel Findings from the NAPLS Cohort

**DOI:** 10.1016/j.psyneuen.2020.104649

**Published:** 2020-05

**Authors:** Alexis E. Cullen, Jean Addington, Carrie E. Bearden, William S. Stone, Larry J. Seidman, Kristin S. Cadenhead, Tyrone D. Cannon, Barbara A. Cornblatt, Daniel H. Mathalon, Thomas H. McGlashan, Diana O. Perkins, Ming T. Tsuang, Scott W. Woods, Elaine F. Walker

**Affiliations:** a Department of Psychosis Studies, Institute of Psychiatry, Psychology & Neuroscience, King’s College London, London, United Kingdom; b Department of Psychiatry, University of Calgary, Calgary, Alberta, Canada; c Department of Psychiatry and Behavioural Sciences and Psychology, UCLA, Los Angeles, United States; d Harvard Medical School, Departments of Psychiatry at Massachusetts Mental Health Center Public Psychiatry Division, Beth Israel Deaconess Medical Center, Massachusetts, General Hospital, Boston, MA, United States; e Department of Psychiatry, University of California, San Diego, CA, United States; f Department of Psychiatry, Yale University, New Haven, Connecticut, United States; g Department of Psychiatry, Zucker Hillside Hospital, Long Island, NY, United States; h Department of Psychiatry, University of California, San Francisco Veterans Affairs Medical Center, San Francisco, CA, United States; i Department of Psychiatry, Yale University, New Haven, CT, United States; j Department of Psychiatry, University of North Carolina, Chapel Hill, NC, United States; k Department of Psychology, Emory University, Atlanta, GA, United States; l Department of Psychiatry, Emory University, Atlanta, GA, United States

**Keywords:** stress adversity, psychosis, schizophrenia, HPA axis responsivity, stressor-cortisol correspondence

## Abstract

Whilst elevations in basal cortisol levels have been reported among individuals at-risk for psychosis, the extent to which this represents hyperresponsivity of the hypothalamic-pituitary-adrenal (HPA) axis to psychosocial stressors encountered in the natural environment is currently unclear. We aimed to examine stressor-cortisol concordance among youth at clinical high-risk (CHR) for psychosis in the North American Prodrome Longitudinal Study 2 (NAPLS 2) and the relationship with clinical outcome. At baseline, CHR (N = 457) and healthy (N = 205) individuals provided salivary cortisol samples and completed daily stressor, life event, and childhood trauma measures. CHR youth were categorised as remitted, symptomatic, progression of positive symptoms, or psychosis conversion at the two-year follow-up. Within-group regression models tested associations between psychosocial stressors and cortisol; standardised beta coefficients (Stβ) were subsequently derived to enable within-group pooling of effect sizes across stressor types. After adjustment for potential confounders, all CHR subgroups reported greater exposure to life events and daily stressors, and more distress in relation to these events, relative to controls. All CHR groups were also more likely to experience childhood trauma; only CHR converters, however, were characterised by elevated basal cortisol. Daily stressor distress was significantly associated with cortisol in controls (β = 0.60, 95% CI: 0.12–1.08) and CHR youth who converted to psychosis (β = 0.91, 95% CI: 0.05–1.78). In controls only, life event exposure was associated with cortisol (β = 0.45, 95% CI: 0.08–0.83). When pooled across stressors, stressor-cortisol concordance was substantially higher among CHR converters (Stβ = 0.26, 95% CI: 0.07 to 0.44) relative to CHR progressed (Stβ = 0.02, 95% CI: -0.11 to 0.15), symptomatic (Stβ = 0.01, 95% CI: -0.11 to 0.12), and remitted groups (Stβ = 0.00, 95% CI: -0.13 to 0.13); however, unexpectedly, healthy controls showed intermediate levels of concordance (Stβ = 0.15, 95% CI: 0.05 to 0.26). In conclusion, whilst all CHR subgroups showed increased psychosocial stress exposure and distress relative to controls, only those who later converted to psychosis were characterised by significantly elevated basal cortisol levels. Moreover, only CHR converters showed a higher magnitude of stressor-cortisol concordance compared to controls, although confidence intervals overlapped considerably between these two groups. These findings do not support the notion that all individuals at CHR for psychosis show HPA hyperresponsiveness to psychosocial stressors. Instead, CHR individuals vary in their response to stressor exposure/distress, perhaps driven by genetic or other vulnerability factors.

## Highlights

• CHR youth showed greater psychosocial stressor exposure and distress than controls.
• Only CHR youth who later developed psychosis had higher basal cortisol at baseline.
• Pooled stressor-cortisol concordance values were highest in CHR converters.
• The degree of concordance distinguished CHR converters from CHR non-converters.
• Concordance in controls was intermediate to CHR converters and non-converters.

## 1 Introduction

Originally formulated over twenty years ago, and recently updated, the neural diathesis-stress model proposes that the hypothalamic-pituitary-adrenal (HPA) axis is the central physiological mechanism linking psychosocial stress to the onset and exacerbation of schizophrenia and related psychotic disorders (Pruessner et al., 2017; Walker et al., 2008; Walker and Diforio, 1997). A central tenet to this model is that individuals with increased vulnerability for psychosis are more sensitive to the effects of psychosocial stressors due to abnormalities within the HPA axis (e.g., HPA hyperactivity/dysregulation or increased glucocorticoid sensitivity) which in turn contribute to dopaminergic and glutamatergic abnormalities that eventually trigger expression of psychotic illness (Pruessner et al., 2017). In support of the model, accumulated evidence indicates that patients with psychosis exhibit elevated basal cortisol relative to healthy controls (Borges et al., 2013; Girshkin et al., 2014; Hubbard and Miller, 2019), but a blunted cortisol awakening response [CAR (Berger et al., 2016; Borges et al., 2013)], the latter thought to represent a distinct HPA axis component, independent of stress-induced cortisol secretion (Boehringer et al., 2015). More recently, these features have been reported among individuals who are at increased risk for psychosis due to clinical features and/or genetic liability (Chaumette et al., 2016; Cullen et al., 2014b; Day et al., 2014; Mittal et al., 2007; Walker et al., 2001; Yildirim et al., 2011). Moreover, at-risk individuals who later develop full psychosis show even greater increases in basal cortisol (Walker et al., 2010; Walker et al., 2013) and pituitary volume (Saunders et al., 2019), suggesting that increased HPA axis activity may signal risk for worsening illness.

In parallel with this research, studies show that at-risk individuals report greater exposure and sensitivity to a range of psychosocial stressors, including major life events, childhood trauma, and minor daily stressors (Cullen et al., 2014a; Fusar-Poli et al., 2017; Kelleher et al., 2008; Mayo et al., 2017; Myin-Germeys et al., 2001; Palmier-Claus et al., 2012; Peh et al., 2019; Tessner et al., 2011; Trotman et al., 2014). However, there has been a paucity of studies examining the concordance between psychosocial stressor exposure/distress and HPA axis function; as such, the extent to which individuals on the psychosis spectrum exhibit ‘abnormal’ HPA axis responses to psychosocial stressors is unclear. That is, the increases in basal cortisol observed in those with, and at-risk for, psychosis may represent either (a) a ‘normal/adaptive’ response to the high levels of psychosocial stressors reported in these populations (i.e., concordance being similar to healthy individuals), or (b) hyperresponsivity of the HPA axis (perhaps due to genetic or early life factors), characterised by an increase in cortisol greater than that expected in a healthy individual (i.e., increased concordance). Alternatively, the elevated basal cortisol levels observed may be partially independent of psychosocial stress exposure/distress (i.e., no concordance), and instead reflect individual-level factors such as genetic predisposition to HPA axis hyperactivity (Hartling et al., 2019; Pagliaccio et al., 2015; Starr et al., 2019) or metabolic abnormalities (e.g., glucose metabolism, which is regulated by the HPA axis), the latter being more common among individuals at clinical high-risk (CHR) for psychosis (Cadenhead et al., 2019), who present features consistent with the prodromal phase of illness.

Two recent studies of at-risk individuals support the ‘increased concordance’ hypothesis: Using the experience sampling method, siblings of psychosis patients showed more pronounced increases in salivary cortisol in response to unpleasant events relative to controls (Collip et al., 2011), whilst a further study reported a stronger association between retrospectively-reported stressful life events and basal cortisol in CHR youth compared to controls (Labad et al., 2015). In contrast, lower (i.e., blunted) cortisol responses during psychosocial stressor tasks have observed in CHR individuals (Pruessner et al., 2013) and young adults with high schizotypy traits (Walter et al., 2018) relative to controls; a pattern consistent with that observed in patients with chronic schizophrenia (Zorn et al., 2017). Together, these findings tentatively suggest that naturally-occurring psychosocial stressors are associated with greater cortisol increases in at-risk individuals compared to healthy controls, whereas the response to experimentally-induced psychosocial stressors is blunted. However, the degree to which HPA axis responses to laboratory-based stressor tasks (which have low ecological validity) are relevant to psychosis aetiology is unclear.

Studying the effect of naturally-occurring stressors on HPA axis function is methodologically complex. Unlike studies using experimentally-induced stressor tasks, the lapse of time between stressor exposure and cortisol measurement may be considerable. Whilst elevations in cortisol levels following stressor exposure appear to decrease over time (Miller et al., 2007), early life events and trauma exposure are associated with HPA dysregulation later in life, suggesting long term effects of stress exposure (Pruessner et al., 2017; Walker et al., 2008; Walker and Diforio, 1997). A related issue is that stress measures and cortisol samples may not be collected on the same day, particularly when studies have large assessment batteries spanning several days. It is possible that day-to-day variations in perceived stress might influence both retrospective reporting of stressful events (and associated distress) and cortisol levels, such that greater concordance is observed when measures are collected on the same day. However, to our knowledge, this has yet to be investigated.

Determining the extent to which HPA axis responsivity in at-risk youth predicts clinical outcome is important, as such work might ultimately help to identify individuals at increased risk of illness progression by virtue of being more sensitive to the effects of psychosocial stress, enabling targeted interventions. Utilising data from the North American Prodrome Longitudinal Study 2 [NAPLS 2, (Addington et al., 2012)] we investigated whether psychosocial stressors, basal cortisol levels, and stressor-cortisol concordance (i.e., the magnitude of association between psychosocial stressors and cortisol) at the baseline assessment differed across healthy controls and CHR subgroups defined on the basis of their clinical presentation at the two-year follow-up (remitted, symptomatic, progression of positive symptoms, and converted to psychosis). Based on previous studies, we hypothesised that CHR youth who later converted to psychosis would show (a) greater exposure and distress in relation to psychosocial stressors, (b) elevated basal cortisol, and (c) higher stressor-cortisol concordance relative to healthy controls; we also anticipated that CHR non-converters would be intermediate to CHR converters subgroups and healthy controls (i.e., converters > positive symptom progression > symptomatic > remitted > controls) on these measures. In all analyses we controlled for a range of potential confounders (age, sex, medication exposure, and cannabis use), and additionally explored the effect of lapse-of-time between assessments on stressor-cortisol concordance.

## 2 Materials and Methods

### 2.1 Sample

NAPLS 2 is a consortium of eight research sites examining CHR youth, the aims and recruitment methods for which are detailed elsewhere (Addington et al., 2012). Briefly, CHR subjects were help-seeking individuals who met criteria for one or more prodromal syndromes: (a) attenuated psychotic symptoms; (b) brief intermittent psychotic symptoms; or (c) substantial functional decline combined with a first-degree relative with a psychotic disorder, or schizotypal personality disorder in individuals younger than 18 years. Prodromal syndromes were assessed using the Criteria of Prodromal Syndromes (COPS), based on the Structured Interview for Prodromal Syndromes [SIPS (McGlashan et al., 2010)], conducted by clinically-trained interviewers; psychiatric diagnoses were determined via the Structured Clinical Interview (SCID) for DSM-IV (First et al., 1995). CHR individuals who had met criteria for an Axis I psychotic disorder were not eligible for inclusion; treatment with antipsychotic medication was permitted provided that full psychotic symptoms were not present at the time of medication commencement. Healthy controls (HC) were recruited from the community and had no personal history or first-degree relative with psychosis and did not meet criteria for any prodromal syndrome. All participants were aged between 12 – 35 years at recruitment. Exclusion criteria for both groups included substance dependence in the past six months, neurological disorder, or full-scale IQ < 70. Non-psychotic psychiatric disorders were permitted in CHR and healthy control groups (Addington et al., 2017).

### 2.2 Procedure

Ethical approval was provided by Institutional Review Boards at each NAPLS site (Addington et al., 2012), all participants provided informed consent or assent. The current sample includes 662 participants for whom variables of interest at baseline (salivary cortisol and at least one of the stress measures examined in the current study) and clinical status at follow-up were available. At baseline, participants provided information on sociodemographic factors and potential confounders, completed stress measures, and collected saliva samples. Baseline assessments were completed over two or more visits. Where possible, saliva was collected on the same day as daily stressor, life event and childhood trauma measures (achieved in 50%, 40%, and 38% of cases, respectively). However, in some in cases (16%), the baseline assessment was interrupted (e.g., due to clinical crises or other life changes) that lead to a substantial delay (> 2 months) in the completion of all measures. In such instances, the remaining baseline measures were collected when the participant was able to return and complete the schedule, with clinical assessments repeated to confirm CHR status. All participants were included in the analysis which accounted for time-lapse between assessments. Prodromal symptoms were assessed via the SIPS at 12- and 24-month follow-up assessments and used to categorise CHR subgroups [see Table 1 for details (Addington et al., 2019; Walker et al., 2013)].

**Table 1:** Categorization of clinical high-risk subgroups at the two-year follow-up.

CHR subgroup	Definition
Remitted	All SIPS positive symptoms rated ≤ 2
Symptomatic	One or more SIPS positive symptoms present in the past 4 weeks, rated 3-5, but with no increase in the past year
Progression of positive symptoms	CHR criteria met with one or more SIPS positive symptoms rated 3-5 and increasing in severity
Converted	A rating of 6 on one or more SIPS positive symptoms

SIPS: Structured Interview for Prodromal Syndromes. Outcome status was determined using information from the final follow-up assessment (in most instances, the 24-month follow-up) with the exception that individuals classified as converters at any follow-up assessment were not reclassified if their symptoms later remitted.

### 2.3 Sociodemographic factors and potential confounders assessed at baseline

Participant date of birth, sex, and ethnicity were assessed via self-report, the latter was subsequently collapsed to a four-level variable (white, black African/African Caribbean, Asian/Middle Eastern, other). Cannabis use was assessed via a structured interview (Buchy et al., 2015). For the purposes of the current investigation we created a binary variable indexing current use (no/yes). Details of all prescribed psychotropic medications (type, name, dosage, and frequency) were obtained at the baseline assessment via self-report, pharmacy records, and/or medical records. Binary variables (no/yes) were created for current antipsychotic use and current psychotropic use (non-antipsychotics), irrespective of type, dose, or data source.

### 2.4 Stress measures

The 58-item, Daily Stress Inventory (Brantley et al., 1987), was used to determine the presence of minor stressors (e.g., ‘Performed poorly at task’, ‘Was ignored by others’) occurring within the past 24 -hs. Participants indicated whether they experienced each stressor and the level of distress elicited by each endorsed stressor (scored on a 7-point scale: 1 ‘occurred but was not stressful’ to 7 ‘caused me to panic’). Total distress scores (range: 0-406) were then divided by the total exposure score (range: 0-58) to obtain an average distress per item score (range: 0-7).

Life events (e.g., ‘End of romantic relationship’, ‘Loss of job’) were assessed via the Psychiatric Epidemiology Research Interview Life Events Scale (Dohrenwend et al., 1978), modified to exclude life events of lesser relevance to youth (e.g., divorce and financial losses) (Trotman et al., 2014). The 59 events can be classified as independent or dependent (of an individual’s influence/behaviour). Interviewers recorded how often each of the 59 events had occurred in the participant’s lifetime and the associated level of distress (scored using the same 7-point scale described above); participants could report multiple exposures to the same event (distress on each occasion also recorded), where the maximum occurrence for any single life event in the NAPLS cohort was four. An average life event distress score (range: 0-7) was derived by dividing the total distress score (potential range: 0-1652) by the total exposure score (potential range: 0-236).

Participants additionally completed the Childhood Trauma and Abuse Scale (Janssen et al., 2004), a semi-structured interview examining experiences of physical, sexual, and psychological abuse, and emotional neglect, occurring prior to age 16 (Addington et al., 2013). Each trauma type was scored as absent/present with a binary variable indexing any form of trauma derived.

### 2.5 Salivary cortisol

At the research session, participants provided three saliva samples (one per hour) with a mean salivary cortisol value subsequently derived when two or more samples were available (participants with only one sample were excluded). The median time of collection for the three samples was 1107 h (range: 0615-1811), 1207 h (range: 0645-1956), and 1300 h (range: 0715-1946), respectively. The mean cortisol value, which is highly correlated with area under the curve (AUC) values (Walker et al., 2010), was computed to provide consistency with previous publications (Walker et al., 2013). Participants were instructed to avoid consumption of caffeine, alcohol, or dairy products after 1900 h on the day before sampling; individuals who reported non-compliance with these instructions were not excluded as previous analyses performed on a subset of the cohort found no association with these variables and cortisol levels (Walker et al., 2013). Use of non-prescription medications (corticosteroids, antihistamines, diet pills, cough/cold medicine) over the past 24 hours was assessed via self-report. Samples were stored at -20 °C, and rapidly thawed and centrifuged prior to assay using a highly sensitive enzyme immunoassay (Salimetrics, State College, Pennsylvania). All samples were assayed in duplicate with intra- and inter-assay coefficients of variation less than 10% and 15%, respectively.

### 2.6 Data analyses

All analyses were performed using Stata Version 15 (StataCorp, 2017). The number of days between stress measure completion and cortisol collection could not be computed for ∼6% of the sample due to missing assessment dates. In such cases, missing values for the three time-lapse variables were imputed using the median number of days (daily stressor and cortisol = 0 days, life events and cortisol = 1 day, childhood trauma and cortisol = 2 days) across the entire sample. The imputed data variables were used in all subsequent analyses.

Ladder and gladder commands were used to identify transformations yielding normally distributed continuous variables. Subsequently, age, cortisol, daily stressor average distress scores, and life event exposure scores were log-transformed, daily stressor exposure scores were square-root transformed, whilst life event average distress scores did not require transformation. There were no transformations that could improve the distribution of the assessment time-lapse variable, therefore a five-level categorical variable was created (1) cortisol collected before stress measurement; (2) assessments completed on the same day; (3) cortisol 1-10 days after stress measurement; (4) cortisol 11-30 days after; and (5) cortisol > 30 days after). Next, we examined correlations between salivary cortisol and sampling variables, namely, time of first sample collection, number of samples collected (two only vs. all three), and use of non-prescription medications in past 24 hours. To remove the influence of relevant factors (those associated with cortisol at the *p* <  0.05 level), cortisol values for the entire sample were regressed on sampling time, cough/cold medication use, and corticosteroid use to obtain standardised residuals. The resulting (normally distributed) variable was used for all subsequent analyses.

Group differences in demographic variables were examined using one-way analysis of variance, Kruskall Wallis, and chi-squared tests. To identify potential confounders, associations among demographic factors, cortisol, and psychosocial stress measures were examined using within-group Pearson’s correlations (for continuous - continuous pairings), biserial correlations (for continuous - categorical pairings), and chi-squared tests (for categorical - categorical pairings). Associations of group status with basal cortisol and psychosocial stressors were next examined, with adjustment for factors that were found in the above steps to be significantly associated with basal cortisol and/or any stressor in any group. Analyses of covariance (ANCOVA), were employed for basal cortisol and continuous stressor measures, with estimated means and standard errors (SE) derived from these models. For trauma exposure, a logistic regression model was used to test the association with group status (testparm command); pairwise comparisons and adjusted trauma prevalence rates and associated SEs for each group were derived from the logistic model. All pairwise comparisons were performed with Sidak correction for multiple testing.

To test whether stressor-cortisol concordance was moderated by time-lapse between assessments, correlations between cortisol and psychosocial stressors were examined within each time-lapse category. Linear regression models were used to test associations between individual psychosocial stressors (predictor variables) and salivary cortisol (outcome variable) in each group. Owing to multicollinearity, each stressor was examined in a separate model; all models were adjusting for potential confounders identified in the above steps. To facilitate comparison of stressor-cortisol concordance across groups, from these adjusted models, we obtained standardised beta coefficients (Stβ) for each psychosocial stress measure and computed SEs for these coefficients [SEStβ = SEβ (Stβ/β)]. Stβ coefficients for each stressor were then pooled using the ‘meta’ command (random effects) to obtain an overall measure of stressor-cortisol concordance.

## 3 Results

### 3.1 Sociodemographic characteristics

Of the 457 CHR individuals included in the current study, 134 (29.3%) showed a remission of CHR symptoms, 130 (28.4%) remained symptomatic, 124 (27.1%) experienced a progression of positive symptoms, and 69 (15.1%) converted to psychosis. The groups did not differ on sex or ethnicity (Table 2); however, there was a relation of follow-up status with age (*F_2,726_* = 5.04, *p* = 0.001), with post-hoc tests indicating that CHR individuals whose symptoms remitted were significantly younger than controls at baseline. Group status was significantly associated with both antipsychotic use (*χ*^2^=36.66, *p* < 0.001) and any other psychotropic use (*χ*^2^= 83.33, *p* = 0.001) at baseline; in both cases, individual chi-squared tests indicated that all four CHR groups were more likely to be treated with medication relative to controls but did not differ from each other. The same pattern was observed for cannabis use, whereby a significant overall association was observed between current use and group status (*χ*^2^=17.61, *p* = 0.001) with individual tests showing that current cannabis use was more common among all CHR groups relative to controls but that the prevalence did not differ across CHR subgroups. Non-parametric Kruskal Wallis (KW) tests performed on the continuous time-lapse variable indicated that the groups did not differ on the lapse-of-time between cortisol collection and daily stressor assessment (KW *χ*^2^= 5.47, *p* = 0.243), cortisol collection and life event assessment (KW *χ*^2^= 4.68, *p* = 0.321), or cortisol collection and trauma assessment (KW *χ*^2^= 8.48, *p* = 0.076).

**Table 2:** Sample Characteristics at Baseline Assessment by Follow-up Status.

	Healthy Controls (n = 205)		CHR Remitted (n = 134)		CHR Symptomatic (n = 130)		CHR Progressed (n = 124)		CHR Converted (n = 69)		Test	*p*
Age (years); mean (SE)^^1^^	20.33	(0.33)	18.31	(0.39)	19.55	(0.38)	19.51	(0.43)	18.52	(0.44)	***F* = 5.04**^a^	**0.001**
Sex (female); n (%)	101	(49.3)	62	(46.3)	55	(42.3)	50	(40.3)	27	(39.1)	*χ^2^*=3.99	0.407
Ethnicity; n (%)												
White	113	(55.1)	77	(57.5)	72	(55.4)	70	(56.5)	35	(50.7)	*χ^2^*=9.97	0.619
Black African/Caribbean	37	(18.1)	19	(14.2)	30	(23.1)	19	(15.3)	11	(15.9)		
Asian/Middle Eastern	23	(11.2)	12	(9.0)	8	(6.2)	9	(7.3)	9	(13.0)		
Other^2^	32	(15.6)	26	(19.4)	20	(15.4)	26	(21.0)	14	(20.3)		
Current antipsychotic use; n (%)	0	(0.0)	23	(17.4)	18	(13.9)	13	(10.7)	12	(17.4)	***χ2* = 36.**66^a,b,c,d^	**<0.001**
Current psychotropic use; n (%)^3^	2	(1.0)	46	(34.8)	48	(36.9)	36	(30.0)	20	(29.0)	***χ2*= 83.33**^a,b,c,d^	**0.001**
Cannabis use ever: n (%)	85	(41.5)	64	(48.1)	76	(58.5)	76	(61.3)	40	(58.8)	***χ2* = 17.**61^b,c,d,f^	**0.001**
No. days between assessments; median (IQR)^4^												
Cortisol and daily stressors	0	(14)	0	(14)	0	(6)	0	(14)	0	(7)	*KW χ^2^*=5.47	0.243
Cortisol and life events	1	(21)	1	(15)	0	(9)	1	(20)	1	(9)	*KW χ^2^*=4.68	0.321
Cortisol and trauma	2	(24)	1	(15)	2	(13)	6	(22.5)	3	(19)	*KW χ^2^*=8.48	0.076

CHR: Clinical high-risk; IQR: interquartile range; KW: Kruskal Wallis (with adjustment for ties).

^1^Descriptive statistics provided for raw (untransformed) age variable with statistical tests performed on log-transformed variable.

^2^Includes First Nations, Central/South American, Native Hawaiian or Pacific Islander, and interracial.

^3^Includes any non-antipsychotic psychotropic medication.

^4^Represents imputed variable (missing data replaced with sample median).

Missing data: current antipsychotic use (n=7); current psychotropic use (n=7). Bold font indicates p < 0.05 for effect of group status. Pair-wise comparisons p <0.05: a Controls vs. CHR Remitted; b Controls vs. CHR Symptomatic; c Controls vs. CHR Progressed; d Controls vs. CHR Converted; e CHR Remitted vs. CHR Symptomatic; f CHR Remitted vs. CHR Progressed; g CHR Remitted vs. CHR Converted; h CHR Symptomatic vs. CHR Progressed; i CHR Symptomatic vs. CHR Converted; j CHR Progressed vs. CHR Converted.

### 3.2 Potential confounders

Age was positively associated with life event exposure and life event distress in controls and all four CHR groups, with daily stressor distress in CHR remitted, symptomatic, and progressed groups, and with cortisol in controls, CHR remitted, and CHR converted groups (*p* <  0.05 for all). Female sex was likewise positively associated with life event exposure in controls, daily stressor exposure in the CHR remitted group, daily stressor distress in CHR symptomatic individuals, and with all five psychosocial stressor measures in the CHR progression of positive symptoms group. In contrast, ethnicity (recoded as white vs. non-white) was not associated with any stress measure or cortisol in any group. With regards to psychotropic medication, antipsychotic use at baseline was negatively correlated with daily stressor exposure in the CHR remitted group and with life event exposure and trauma in the CHR progressed group, but positively associated with daily stressor distress in CHR individuals who later converted to psychosis; similarly, other psychotropic medication was negatively correlated with daily stressor exposure in the remitted group but positively correlated with daily stressor and life event distress variables in the converter group. Current cannabis use was associated positively with daily stressor exposure, life event distress, and trauma in the control group and with life event exposure in remitted and symptomatic groups.

The above analyses identified the following baseline factors as potential confounders in the relationship between stress and cortisol: age, sex, current antipsychotic use, current other psychotropic medication use, and current cannabis use. However, owing to multicollinearity issues (current antipsychotic and other psychotropic medication use were strongly associated: *χ*^2^= 94.89, *p* < 0.001), all models included antipsychotic use only as a covariate, with sensitivity analyses performed using other psychotropic medication in place of antipsychotic use. We were additionally concerned that controlling for current cannabis use might obscure important relationships between stress and cortisol (Miller and Chapman, 2001), given that recent evidence indicates that stress can precipitate cannabis use in healthy and clinical samples (Hyman and Sinha, 2009), and therefore included cannabis use as a covariate in sensitivity analyses only.

### 3.3 Group differences in cortisol and psychosocial stressors

ANCOVAs (basal cortisol, continuous stress measures) and logistic regression (trauma exposure) indicated significant main effects of group status on basal cortisol (*p* =  0.013) and all stress measures (*p* <  0.001) after adjustment for age, sex, and antipsychotic use at baseline (Table 3). Post-hoc comparisons (Sidak-corrected) indicated that only CHR convertors were characterised by elevated basal cortisol compared to controls (*t* = 3.19, *p* = 0.015), no other group differences were observed. With regards to continuous stress measures (daily stressor exposure and distress scores and life event exposure and distress scores), all four CHR subgroups were characterised by significantly higher scores relative to controls; for daily stressor exposure only, symptomatic, progressed, and converted groups also showed significantly higher scores relative to remitted CHR youth. To confirm that the greater exposure to life events observed in CHR subgroups was not simply due to events that could be caused by illness, we additionally compared groups on exposure to independent life events (data not shown) and observed a significant main effect of group status (*F* = 10.86, *p* < 0.001). Post-hoc tests indicated that all CHR subgroups, except for the remitted group, reported increased exposure to independent life events compared to controls. Finally, childhood trauma was more common in all CHR groups compared to controls but did not distinguish among CHR subgroups. All results were largely unchanged when (a) cannabis use was included as an additional covariate and (b) other psychotropic medication use was additionally included in place of antipsychotic medication, with the exception that CHR remitted youth no longer showed significantly greater life event exposure compared to controls.

**Table 3:** Basal Cortisol and Psychosocial Stressors by Follow-up Status Adjusted for Age, Sex, and Antipsychotic Use at Baseline

	Controls (n = 205)		CHR Remitted (n = 134)		CHR Symptomatic (n = 130)		CHR Progressed (n = 124)		CHR Converted (n = 69)		Test^1^	***p***
Salivary cortisol (raw); mean (SE)	16.56	(0.76)	15.22	(0.80)	18.58	(1.10)	17.24	(0.99)	19.36	(1.42)	***F* = 3.21**^^d^^	**0.013**
Salivary cortisol (adjusted); mean (SE)	−0.16	(0.07)	−0.05	(0.09)	0.12	(0.09)	−0.04	(0.09)	0.30	(0.12)		
Daily stressor exposure (raw); mean (SE)	14.18	(0.67)	17.47	(0.93)	23.30	(1.10)	23.84	(1.12)	25.94	(1.75)	***F* = 27.22**^a,b,c,d,e,f,g^	**<0.001**
Daily stressor exposure (adjusted); mean (SE)	3.48	(0.10)	4.02	(0.12)	4.65	(0.12)	4.71	(0.12)	4.91	(0.16)		
Daily stressor distress (raw); mean (SE)	1.95	(0.06)	2.84	(0.10)	3.20	(0.11)	3.04	(0.11)	3.21	(0.17)	***F*= 39.62**^a,b,c,d^	**<0.001**
Daily stressor distress (adjusted); mean (SE)	0.57	(0.03)	0.97	(0.04)	1.08	(0.04)	1.02	(0.04)	1.08	(0.05)		
Life event exposure (raw); mean (SE)	20.73	(0.88)	25.40	(2.45)	33.02	(2.80)	26.05	(1.65)	26.67	(2.51)	***F* = 12.40**^a,b,c,d^	**<0.001**
Life event exposure (adjusted); mean (SE)	2.78	(0.04)	3.01	(0.05)	3.21	(0.05)	3.06	(0.05)	3.18	(0.07)		
Life event distress (raw); mean (SE)	2.74	(0.07)	3.40	(0.10)	3.81	(0.10)	3.66	(0.10)	3.82	(0.15)	***F* = 32.48**^a,b,c,d^	**<0.001**
Life event distress (adjusted); mean (SE)	2.68	(0.08)	3.46	(0.09)	3.80	(0.09)	3.67	(0.09)	3.88	(0.13)		
Trauma history (raw); n (%)	27	(14.8)	63	(51.6)	67	(54.5)	72	(63.7)	40	(60.6)	***χ2*= 89.10**^a,b,c,d^	**<0.001**
Trauma history (adjusted); % (SE)	13.49	(2.43)	53.74	(4.47)	54.74	(4.34)	64.17	(4.39)	62.76	(5.73)		

CHR: Clinical high-risk; SE: standard error.

^1^For all continuous variables, raw (untransformed variable) and adjusted (transformed variable, for all except life event distress, adjusted for age, sex, current antipsychotic medication) descriptive statistics provided. For categorical variables, raw (absolute n and %) and adjusted (age-, sex- and antipsychotic-adjusted proportions and SE) descriptive statistics provided.

Missing data: daily stressor exposure (n=17); daily stressor distress (n=19); life event exposure (n=14); life event distress (n=30), trauma history (n=56). Bold font indicates p < 0.05 for effect of group status. Pair-wise comparisons with Sidak corrections for multiple testing p <0.05: a Controls vs. CHR Remitted; b Controls vs. CHR Symptomatic; c Controls vs. CHR Progressed; d Controls vs. CHR Converted; e CHR Remitted vs. CHR Symptomatic; f CHR Remitted vs. CHR Progressed; g CHR Remitted vs. CHR Converted; h CHR Symptomatic vs. CHR Progressed; i CHR Symptomatic vs. CHR Converted; j CHR Progressed vs. CHR Converted.

### 3.4 Stressor-cortisol concordance by time lapse between assessments

**Fig. 1:**
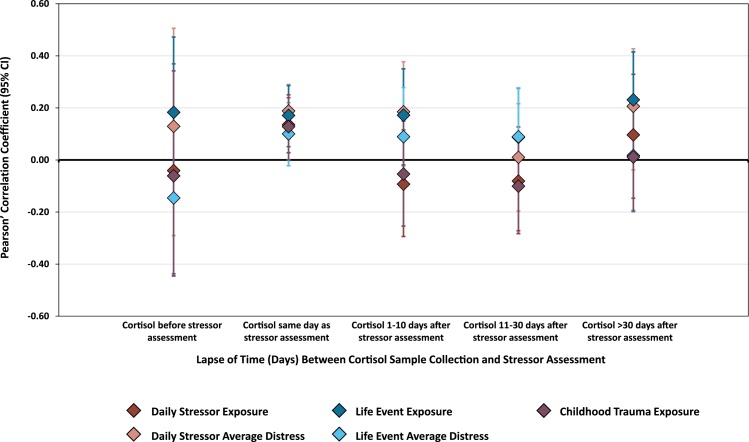
Stressor-cortisol concordance by assessment time-lapse across the total sample. Correlations between psychosocial stressors and basal cortisol according to lapse of time between stressor assessment and salivary cortisol collection (days). Number of participants per time-lapse period for (a) daily stressor exposure/distress and cortisol: cortisol before stressor (n = 26), same day (n = 381), cortisol 1-10 days after (n = 91), cortisol 11-30 days after (n = 94), cortisol > 30 days after (n = 70); (b) life event exposure/distress and cortisol: cortisol before stressor (n = 41), same day (n = 264), cortisol 1-10 days after (n = 152), cortisol 11-30 days after (n = 107), cortisol > 30 days after (n = 98); and (c) trauma exposure and cortisol: cortisol before stressor (n = 28), same day (n = 251), cortisol 1-10 days after (n = 144), cortisol 11-30 days after (n = 135), cortisol > 30 days after (n = 104).

In line with predictions, stressor-cortisol concordance varied according to the lapse-of-time between assessments (see Fig. 1): When acquired on the same day as saliva sampling, all stress measures showed significant, positive correlations with cortisol (*p <* 0.05), except for life event distress scores which were positively correlated but not significantly. In contrast, stress measures were not significantly correlated with cortisol in any other time-lapse category except for life event exposure and cortisol which were positively associated when the lapse-of-time was 31 days or longer. To account for the moderating effect of time-lapse between assessments, interaction terms (stress*time-lapse) were additionally included in subsequent regression models.

### 3.5 Stressor-cortisol concordance by clinical status at follow-up

**Fig. 2:**
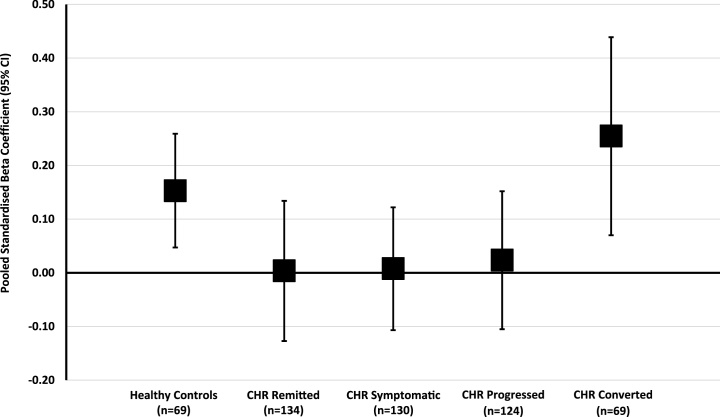
Pooled standardised beta coefficients (with 95% confidence intervals), representing the magnit ude of stressor-cortisol concordance, by group status at follow-up.

Within-group linear regression analyses were performed (Table 4) to examine stressor-cortisol concordance after adjustment for potential confounders and the interaction between stressors and lapse-of-time between assessments. Daily stressor distress was significantly associated with cortisol in both controls (β = 0.60, 95% CI: 0.12 to 1.08) and CHR converters (β = 0.91, 95% CI: 0.05 to 1.78). In the control group only, greater life event exposure was associated with higher cortisol ( β= 0.45, 95% CI: 0.08 to 0.83). In contrast, none of the stress measures were significantly associated with cortisol in the CHR remitted, symptomatic, or progressed subgroups. Sensitivity analyses (data not shown) indicated that there was no change to the overall pattern of findings when cannabis use was included as an additional covariate or when any psychotropic use was used in place of antipsychotic use. Moreover, when we additionally adjusted all models for exposure to dependent life events, results were unchanged. When Stβ coefficients were pooled across all five stress measures, stressor-cortisol concordance was highest in the CHR converted group (Stβ = 0.26, 95% CI: 0.07 to 0.44), intermediate in controls (Stβ = 0.15, 95% CI: 0.05 to 0.26), and lowest in the CHR progressed (Stβ = 0.02, 95% CI: -0.11 to 0.15), symptomatic (St = 0.01, 95% CI: -0.11 to 0.12), and remitted groups (Stβ = 0.00, 95% CI: -0.13 to 0.13). Fig. 2 illustrates the pattern of pooled stressor-cortisol across groups; whilst confidence intervals for CHR converted and healthy control groups overlapped substantially, the former were clearly distinguished from non-converted CHR subgroups (Cumming, 2009).

**Table 4:** Results of Multivariable Linear Regression Analyses Examining the Effect of Psychosocial Stressors on Basal Cortisol by Follow-up Status

	Controls (n = 205)			CHR Remitted (n = 134)			CHR Symptomatic (n = 130)			CHR Progressed (n = 124)			CHR Converted (n = 69)		
	β	(95% CI)	*p*	β	(95% CI)	*p*	β	(95% CI)	*p*	β	(95% CI)	*p*	β	(95% CI)	*p*
Daily stressor exposure	0.01	(-0.16–0.17)	0.946	0.04	(-0.17–0.25)	0.693	0.10	(-0.06–0.26)	0.241	0.04	(-0.18–0.26)	0.714	0.12	(-0.13–0.36)	0.331
Daily stressor distress	**0.60**	**(0.12–1.08)**	**0.014**	0.00	(-0.56–0.55)	0.986	0.17	(-0.36–0.70)	0.536	0.18	(-0.41–0.77)	0.552	**0.91**	**(0.05–1.78)**	**0.038**
Life event exposure	**0.45**	**(0.08–0.83)**	**0.017**	−0.34	(-0.78–0.11)	0.134	−0.06	(-0.56–0.44)	0.819	−0.10	(-0.49–0.30)	0.623	0.24	(-0.42–0.90)	0.462
Life event distress	0.06	(-0.19–0.30)	0.643	−0.03	(-0.28–0.22)	0.814	−0.01	(-0.28–0.25)	0.921	0.02	(-0.23–0.26)	0.889	0.11	(-0.28–0.51)	0.567
Trauma exposure	0.56	(-0.07–1.20)	0.081	0.33	(-0.19–0.85)	0.205	−0.46	(-1.03–0.11)	0.116	0.09	(-0.63–0.82)	0.800	0.77	(-0.15–1.68)	0.098

CHR: Clinical high-risk; β: unstandardized beta coefficient; CI: confidence interval. All models adjusted for time lapse between psychosocial stress assessment and cortisol measurement, interaction between time lapse and stress variable, age, sex, and current antipsychotic medication use at baseline. Missing data: daily stressor exposure (n = 17); daily stressor distress (n = 19); life event exposure (n = 14); life event distress (n = 30), trauma history (n = 56). Bold font indicates *p* <  0.05 for effect of psychosocial stressor on basal cortisol.

## 4 Discussion

The current study aimed to further characterise the nature of HPA axis abnormalities among individuals at-risk for psychosis by examining psychosocial stressors, basal cortisol, and the concordance between these measures in a large sample of CHR youth categorised according to clinical status at the two-year follow-up. In line with hypotheses, all CHR groups were characterised by significantly greater psychosocial stressor exposure and distress relative to healthy controls; however, only those who converted to psychosis demonstrated elevated basal cortisol levels. In contrast to expectations, whilst CHR converters showed the greatest degree of stressor-cortisol concordance when pooled across stressors, confidence intervals substantially overlapped with the control group; moreover, the degree of concordance among CHR youth who remitted, remained symptomatic, or whose positive symptoms had progressed at follow-up was lower than that observed in the control group.

After adjustment for potential confounders (including dependent life events) and correction for multiple testing, only CHR converters showed elevated basal cortisol relative to healthy controls. This finding cannot be simply attributed to greater stressor exposure or distress experienced by CHR converters relative to controls, as these features characterised all CHR subgroups. This elevation might instead reflect an amplification of the normative adolescent increase in cortisol secretion (Apter et al., 1979; Marceau et al., 2015), or metabolic abnormalities [more common among CHR youth (Cadenhead et al., 2019)], independent of stress exposure. Consistent with a recent meta-analysis (Chaumette et al., 2016), pairwise comparisons showed that basal cortisol levels did not distinguish CHR converters from CHR non-converters. Whilst this suggests that within the CHR population, baseline cortisol levels do not signal risk for psychosis transition, repeated measurement of cortisol is needed to determine whether longitudinal increases predict poorer outcomes in this group. Moreover, it should be assumed that at 2-year follow-up there are some false negative cases in the CHR non-converted groups (Ziermans et al., 2014), and thus differences between converters and non-converters may increase with a longer follow-up period.

We predicted that the degree of stressor-cortisol concordance, when pooled across stressors, would increase in parallel with the level of symptom expression at follow-up (i.e., controls < remitted < symptomatic < progression of psychotic symptoms < converters). Whilst the highest degree of concordance was indeed observed among the CHR converters, the control group was intermediate, and pooled beta coefficients in the three non-converted CHR subgroups were approximately zero (indicating no significant association between stressor measures and basal cortisol). Moreover, confidence intervals for pooled stressor-cortisol concordance estimates for all CHR subgroups (particularly the converter group) showed a high degree of overlap with the control group (Fig. 2); it has been proposed that for many effect sizes, confidence intervals overlapping by greater than 50% suggests that effect sizes are not significantly different (Cumming, 2009). Thus, none of the CHR subgroups showed significant hyper- or hypo-responsivity of the HPA axis in response to psychosocial stressors encountered in the natural environment when compared to controls. Stressor-cortisol concordance was, however, substantially higher among CHR converters compared to all other CHR subgroup (i.e., confidence intervals appeared to overlap < 50%). This finding is consistent with the only previous study to examine the relationship between stressor-cortisol concordance and outcome status in CHR individuals: Labad and colleagues similarly reported a moderate-to-strong (but not statistically significant) correlation between salivary cortisol and stressful life events among those who later transitioned to psychosis, but only a weak correlation in the non-transitioned CHR group (Labad et al., 2015).

Overall, we found few significant associations between individual stressors and basal cortisol across all groups (Table 4). Whilst this could be due to the HPA axis and/or stress measures employed, previous studies of at-risk youth which have used different measures have likewise found inconsistent associations between stress and cortisol (Cullen et al., 2014b; Labad et al., 2015; Thompson et al., 2007). Similarly, in healthy subjects, correlations between self-reported stress and cortisol have not been observed (Cummins and Gevirtz, 1993; Vedhara et al., 2003). Although the exact mechanisms underlying HPA responsivity to stress are unknown (and likely complex), it has been demonstrated that there are individual differences in responsivity that are partially determined by genetic variants (e.g., FKBP5, CRHR1, NR3C1, NR3C2) that modify the effect of acute and chronic stress/trauma on cortisol levels in healthy adolescents and adults (Hartling et al., 2019; Starr et al., 2019; Utge et al., 2018) and patients with psychosis (Mondelli and Ciufolini, 2017). Thus, genetic and other vulnerability factors are likely responsible for the different patterns of association between stressors and cortisol that we observed across both individual stressor types and, when pooled across stressors, CHR subgroups.

### 4.1 Limitations

Despite the large overall sample size, individual CHR subgroups were notably smaller (particularly the converted group) thereby reducing our ability to detect statistically significant associations between psychosocial stressors and cortisol. Conversely, as we did not adjust for multiple comparisons in our primary analyses examining stressor-cortisol concordance, some significant associations may have arisen by chance. However, we tested specific *a priori* hypotheses and were largely interested in the overall pattern of stressor-cortisol concordance rather than statistical significance. Moreover, we adjusted for a range of potential confounders which, had we not accounted for these variables, would have led to spurious associations. A further limitation is that a small proportion of participants (16%), experienced a long delay (> two months) between baseline assessment visits, which led to a large lapse-of-time between completion of psychosocial stressor assessments and cortisol collection. Including these participants in the analyses increased statistical power to test the moderating effect of time-lapse on stressor-cortisol concordance. One major limitation is that we examined only three stressor types. There are a range of other stressors relevant to psychosis that might conceivably impact on HPA axis function (e.g., urbanicity, neighbourhood cohesion, and socioeconomic deprivation); it is possible that examining a wider range of stressors might yield different patterns of stressor-cortisol concordance across CHR subgroups. Similarly, our findings are specific to basal salivary cortisol, other measures (e.g., plasma cortisol or salivary diurnal or awakening cortisol profiles) may have produced different results.

Whilst the aim of our study was to examine the relationship between baseline features (psychosocial stressors, basal cortisol, and stressor-cortisol concordance) and subsequent outcome (based on progression to psychosis), it is important to note that there are limitations with this approach. First, we did not account psychosocial stressors and other confounding factors/events that may have occurred in the time between baseline and follow-up. Indeed, it is possible that stressor-cortisol concordance at follow-up does in fact distinguish between CHR subgroups, but that our measure at baseline was too distal to outcome. Second, CHR individuals are at elevated risk for a wide range of psychiatric disorders, particularly depression and anxiety (Addington et al., 2017; Addington et al., 2019), and so worsening of prodromal symptoms/transition to psychosis is only one of several potential outcome measures, all of which will inevitably involve more false negatives the shorter the follow-up period. Indeed, a recent study suggested that well-established risk factors are better at predicting poor functioning in CHR populations than transition to psychosis (Zhang et al., 2019). The extent to which stressor-cortisol concordance at baseline is associated with other non-psychotic disorders and functioning at follow-up is therefore warranted.

### 4.2 Directions for future research

We assessed HPA axis function using basal salivary cortisol collected in the laboratory, as it is more reliable and, unlike home sampling methods, unlikely to be influenced by confounding factors such as exercise (Hill et al., 2008; Laceulle et al., 2015). However, meta-analytic evidence indicates that the effect of chronic stress on cortisol varies across cortisol measures; whilst diurnal (overall daily output) cortisol, afternoon/evening cortisol, and the CARi (increase in cortisol following awakening) are elevated following chronic stress, basal morning levels are lower and the diurnal rhythm appears to be flatter (Chida and Steptoe, 2009; Miller et al., 2007). Employing alternative cortisol measures might therefore reveal different patterns of stressor-cortisol concordance across CHR individuals and controls. Indeed, using a home sampling procedure, Cullen and colleagues reported a negative correlation between the CARi and negative life event distress in at-risk children with a family history of schizophrenia but a positive correlation in typically-developing children (Cullen et al., 2014b), whilst a study of adults found that diurnal cortisol was associated negatively with stressful life event exposure in first-episode psychosis patients, most of whom were receiving antipsychotic medication, but positively in controls (Mondelli et al., 2010). Thus, employing multiple measures of cortisol may be more informative than basal cortisol alone and enable the identification of dissociated relationships in at-risk individuals/psychosis patients and healthy controls.

Cortisol output is not, however, the only method of assessing HPA axis function. In addition to endocrine measurement (which extends to other HPA-axis hormones, e.g., adrenocorticotropic hormone) neuroimaging can be used to determine pituitary and hippocampal volume (key structures involved in mediating HPA axis function) and density, distribution and/or affinity of glucocorticoid/mineralocorticoid receptors. The latter is particularly important as these receptors mediate the effects of glucocorticoids on cellular targets. As a related point, future studies are warranted to investigate glucocorticoid sensitisation (i.e., the responsiveness to increased glucocorticoids over time), in CHR youth, as this may have implications not only for the HPA axis, but also the immune system (Cain and Cidlowski, 2017), and dopamine levels (Lefevre et al., 2017). Thus, studies employing multiple methodological approaches, including genetic profiling, neuroimaging, and endocrine measurement, may be needed to adequately investigate the extent to which individuals at-risk for psychosis are characterised by increased HPA axis sensitivity.

Our findings have other implications for future research examining HPA axis responsivity in at-risk individuals. First, we observed that the lapse-of-time between completion of stress measures and cortisol collection moderated stressor-cortisol concordance (with minor exceptions, significant relationships were observed only when measures were collected on the same day). Whilst we anticipated this pattern for daily stressors occurring within the past 24 hours, the findings for life events and childhood trauma were not predicted as these events did not occur on the day of measurement. It is plausible that reporting these events in the research environment is itself a stressful experience for some participants, and that it elicits a cortisol elevation and thus a relationship between stressor exposure and cortisol. These findings highlight the importance of adjusting for the interaction between stressors and time-lapse between assessments when examining stressor-cortisol concordance. Second, participant sex was identified as a potential confounder. The updated neural diathesis-stress model noted sex differences to be an important area for future research (Pruessner et al., 2017), whilst it was beyond the scope of the current study to explore whether sex modified the degree of stressor-cortisol concordance, future studies should investigate this possibility. Finally, whilst we defined CHR outcome status on the basis of attenuated positive symptoms and transition to psychosis, as noted above, there has been recent acknowledgement of the need to examine a broader range of outcomes, including, levels of social and role functioning, non-psychotic disorders, and negative symptoms (Addington et al., 2011; Yung et al., 2019). Future studies might therefore examine whether stressor-cortisol concordance is associated with these outcomes at follow-up.

### 4.3 Conclusions

The original neural diathesis-stress model of schizophrenia (Walker and Diforio, 1997) and subsequent revisions of this hypothesis (Pruessner et al., 2017; Walker et al., 2008) hypothesized that heightened HPA axis activity among those at elevated risk for psychosis could be stress-induced, a manifestation of hippocampal dysfunction or glucocorticoid receptor abnormalities, or genetically determined. In the current study, we tested the first of these possibilities and conclude that the elevation in basal cortisol levels among CHR converters is not simply due to increased psychosocial stressor exposure and distress, as the latter was common to all CHR subgroups. Our analyses did, however, show that naturally-occurring psychosocial stressors were more strongly associated with basal cortisol levels in CHR converters compared to CHR non-converters (perhaps driven by genetic factors), although the degree of concordance did not appear to differ significantly in converters and healthy controls. Given the novelty of this investigation, this pattern of findings warrants further investigation in other at-risk populations.

## Declaration of Competing Interest

None to report for all authors.

## Author contributions

The NAPLS Lead Investigators (J.A., C.E.B., W.S.S., L.J.S., K.S.C., T.D.C., B.A.C., D.H.M., T.H.M., D.O.P., M.T.T., S.W.W., E.F.W) were responsible for the design of the NAPLS project and supervision of all aspects of data collection. A.E.C. and E.F.W. conceived the design for the present study, A.E.C. conducted the analyses and wrote the manuscript. All authors contributed to manuscript revisions and provided intellectual input.
